# Efficacy of Photodynamic Therapy in Chronic Central Serous Chorioretinopathy: A Retrospective Analysis at Hanusch Hospital, Vienna

**DOI:** 10.1055/a-2772-8827

**Published:** 2026-02-10

**Authors:** Christoph Spartalis, Christoph Hackl, Stephan Radda, Oliver Findl

**Affiliations:** Ophthalmology, Hanusch Hospital, Vienna, Austria

**Keywords:** PDT, retina, cCSC, CSC, CRCS, PDT, Netzhaut, cCSC, CSC, CRCS

## Abstract

**Background** Chronic central serous chorioretinopathy (cCSC) is an exudative maculopathy characterised by persistent subretinal fluid and potential visual deterioration. Photodynamic therapy (PDT) with verteporfin is an established treatment option, particularly in refractory or chronic cases. This retrospective, single centre study aimed to evaluate the functional and morphological efficacy of PDT in patients with cCSC at Hanusch Hospital, Vienna.

**Patients/Materials and Methods** We analysed 49 eyes of 38 patients who underwent PDT between 2020 and 2025. Best-corrected visual acuity (decimal), central retinal thickness (CRT, µm), and subretinal fluid (SRF, µm) were assessed using spectral domain OCT before and after treatment. Statistical analyses included the Shapiro-Wilk test for normality and the Wilcoxon signed-rank test for paired samples. Subgroup analyses were conducted for repeat PDT (Re-PDT) and secondary choroidal neovascularisation (CNV).

**Results** Overall, PDT led to a significant improvement in visual acuity (p = 0.041) and a highly significant reduction in both CRT and SRF (each p < 0.001). In the Re-PDT subgroup (n = 6), significant morphological improvement (p = 0.028 for CRT and SRF) was observed without a statistically significant gain in vision (p = 0.109). No significant therapeutic effect was found in patients with secondary CNV. There was a significant negative correlation between visual acuity and CRT (r = − 0.42; p = 0.005) and between visual acuity and SRF (r = − 0.37; p = 0.012).

**Conclusion** PDT appears to be an effective treatment for achieving anatomical and functional stabilisation in cCSC. The limited visual improvement in Re-PDT and CNV subgroups underscores the importance of early treatment and tailored decision-making in cases of secondary neovascularisation.

## Zusammenfassung

**Hintergrund** Die chronische zentrale seröse Chorioretinopathie (cCSC) ist eine exsudative Makulopathie, die durch persistierende subretinale Flüssigkeit und potenzielle Visusverschlechterung gekennzeichnet ist. Die photodynamische Therapie (PDT) mit Verteporfin stellt insbesondere bei chronischen oder therapieresistenten Verläufen eine etablierte Behandlungsoption dar. Ziel dieser retrospektiven monozentrischen Studie war es, die funktionelle und morphologische Wirksamkeit der Half-Dose-PDT bei Patienten mit cCSC zu evaluieren.

**Patienten/Material und Methoden** In die Analyse wurden 49 Augen von 38 Patienten eingeschlossen, die zwischen 2020 und 2025 eine Half-Dose-PDT erhalten hatten. Die bestkorrigierte Sehschärfe (BCVA), die zentrale Netzhautdicke (CRT) sowie die Menge subretinaler Flüssigkeit (SRF) wurden mittels Spectral-Domain-OCT vor und nach der Behandlung erhoben. Zur statistischen Auswertung wurden der Shapiro-Wilk-Test zur Prüfung der Normalverteilung sowie der Wilcoxon-Vorzeichen-Rang-Test für verbundene Stichproben verwendet. Explorative Subgruppenanalysen wurden für Re-PDT-Behandlungen sowie Fälle mit sekundärer choroidaler Neovaskularisation (CNV) durchgeführt.

**Ergebnisse** Die Half-Dose-PDT führte zu einer signifikanten Verbesserung der BCVA (p = 0.041) sowie zu einer hochsignifikanten Reduktion von CRT und SRF (jeweils p < 0.001). In der explorativen Re-PDT-Subgruppe zeigte sich eine signifikante morphologische Besserung (CRT und SRF jeweils p = 0.028), jedoch keine signifikante Veränderung der BCVA (p = 0.109). Bei Patienten mit sekundärer CNV ließ sich kein therapeutischer Nutzen nachweisen. Die BCVA korrelierte negativ mit CRT (r = − 0.339; p = 0.033) und SRF (r = − 0.511; p < 0.001).

**Schlussfolgerung** Die Half-Dose-PDT zeigt bei cCSC eine deutliche anatomische und funktionelle Wirksamkeit. Die explorativen Subgruppenanalysen unterstreichen die Bedeutung einer frühzeitigen Therapie und einer individualisierten Entscheidungsfindung. Aufgrund des retrospektiven monozentrischen Studiendesigns ohne Kontrollgruppe sind kausale Schlussfolgerungen jedoch nur eingeschränkt möglich.

## Introduction

Central serous chorioretinopathy (CSC) is a disease of the posterior segment of the eye, characterized by a central serous detachment of the neurosensory retina in the macular region. It primarily affects men between the ages of 30 and 60 and occurs approximately six times more frequently in men than in women 1 . The chronic form of the disease (cCSC), defined by the persistence of subretinal fluid for more than three months, is of particular clinical concern due to the risk of permanent functional impairment and structural damage 2 .

Known risk factors include psychological stress, corticosteroid use, type A personality traits, sleep disturbances, systemic hypertension, smoking and shift work 3 , 4 . Genetic predispositions have also been suggested. Bilateral involvement has been described and may indicate systemic contributors 5 . These risk factors are believed to influence the pathogenesis via hormonal or vascular mechanisms affecting choroidal blood flow and vascular permeability. Histologically, choroidal vessel dilation and dysfunction of the retinal pigment epithelium (RPE) along with increased choroidal thickness are key features 6 , 7 . Recent evidence further implicates increased scleral thickness and asymmetric vortex veins as potential contributors to impaired venous outflow and choroidal congestion, supporting the concept of pachychoroid syndrome and venous overload choroidopathy 8 9 10 .

Despite extensive research, a unified definition of chronic CSC remains lacking, which hinders comparability across studies. Clinical interpretation also varies significantly due to individual differences in disease duration, symptom severity, and natural progression.

Photodynamic therapy (PDT) is currently the most well-studied interventional treatment option for cCSC. It involves intravenous administration of verteporfin, a light-activated photosensitizer that accumulates selectively in pathologically altered choroidal vessels. Subsequent laser irradiation at 689 nm triggers a photochemical reaction that leads to remodeling of the choroidal vasculature and reduced choroidal permeability 11 .

Several clinical trials have shown that the so-called “half-dose” or “half-fluence” PDT regimens achieve good efficacy with reduced risk of side effects 12 , 13 . Both strategies have demonstrated comparable outcomes in terms of anatomical and functional recovery in randomized trials 14 . Key outcome measures include normalization of central retinal thickness (CRT) and resolution of subretinal fluid (SRF). Notably, the PLACE trial demonstrated significantly greater efficacy of PDT over subthreshold micropulse laser therapy regarding anatomical recovery and functional stabilization 12 .

## Patients/Materials and Methods

This retrospective single-center data analysis was conducted at Hanusch Hospital, Vienna. All patients with clinically and imaging-confirmed (optical coherence tomography, indocyanine green angiography [ICG]) cCSC who received half-dose PDT between January 2020 and May 2025 were included. cCSC was defined as the persistence of subretinal fluid for at least three months.

### Photodynamic therapy protocol

All patients underwent ICG–guided half-dose PDT using verteporfin (Visudyne, Novartis, Switzerland). The verteporfin dose was 3 mg/m² body surface area, infused intravenously over 10 min, followed by laser activation at 689 nm using a fluence of 50 J/cm², irradiance of 600 mW/cm², and an exposure time of 83 s. The treatment spot size was defined according to the area of choroidal hyperpermeability observed on ICG angiography, usually encompassing the leakage area with a 500 µm safety margin. Re-PDT was considered in cases of persistent or recurrent subretinal fluid beyond 3 months after the initial treatment, provided that ICG angiography demonstrated residual or re-emerging zones of hyperpermeability.

The study included both initial and repeated ICG guided PDT treatments. This study was conducted in accordance with the Declaration of Helsinki and approved by the institutional ethics committee (Approval Number MA15-25-144-VK).

The following parameters were recorded: age, sex, and per eye: best-corrected visual acuity (in decimal notation), central retinal thickness (CRT in µm), and amount of subretinal fluid (SRF in µm) before and after treatment, the median disease duration prior treatment, the mean photodynamically treated area. Data were collected using spectral-domain OCT and evaluated in a standardized manner. Additionally, the presence of choroidal neovascularization (CNV) was documented.

All statistical analyses were performed using IBM SPSS Statistics (version 29.0, IBM Corp., Armonk, NY, USA). Graphs and visualizations were generated with GraphPad Prism (version 10.0, GraphPad Software, San Diego, CA, USA), Microsoft Excel (version 16.77, Microsoft Corp., Redmond, WA, USA), and Matplotlib (Python 3.11, Python Software Foundation), and finalized for figure preparation using Microsoft PowerPoint (version 16.77, Microsoft Corp., Redmond, WA, USA).

The Shapiro-Wilk test was applied to assess normal distribution. For comparison of pre- and post-treatment values for visual acuity, CRT, and SRF, the Wilcoxon signed-rank test for paired samples was used. Due to the limited number of cases in the Re-PDT and CNV subgroups, the Wilcoxon signed-rank test was applied using exact significance calculation to account for small-sample bias. Spearman rank correlations were also computed to evaluate the relationship between functional (visual acuity) and morphological (CRT, SRF) parameters. Gender-specific differences were assessed using the Mann-Whitney U test, and a Spearman correlation was used to analyze the relationship between age and treatment outcome. Results with a p-value < 0.05 were considered statistically significant.

To account for potential intra-patient correlation in bilateral cases, a sensitivity analysis including only one eye per patient (the eye with poorer baseline visual acuity) was conducted. The Wilcoxon signed-rank test was repeated for BCVA, CRT, and SRF to confirm the robustness of the main findings.

**Fig. 1:**
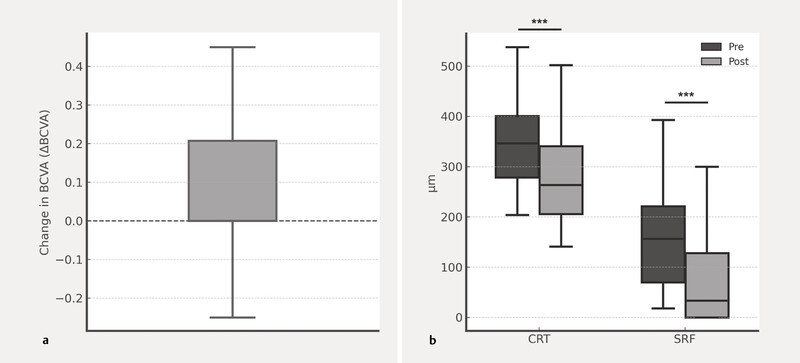
**a** Change in best-corrected visual acuity (BCVA) before and after photodynamic therapy (PDT) in the overall cohort. Boxplot illustrating a statistically significant improvement in visual acuity following PDT (p = 0.041). **b** Central retinal thickness (CRT) and subretinal fluid (SRF) before and after PDT in the overall cohort. Boxplots showing a significant reduction in CRT and SRF after treatment (both p < 0.001), indicating robust anatomical response.

## Results

A total of 49 treated eyes from 38 patients were included in the analysis. Both functional (visual acuity) and morphological (CRT, SRF) parameters were systematically documented and compared before and after PDT. Baseline demographic and clinical characteristics of the study cohort, including disease duration, bilateral cases, prior treatments, and systemic risk factors, are summarized in Table 1 .

**Table:** **Table 1** Baseline demographic and clinical characteristics of the study cohort.

Parameter	Value
Number of patients (eyes)	38 (49)
Gender (M/F)	32/17
Mean age (years ± SD, range)	63.5 ± 13.6 (34 – 88)
Median disease duration (months, range)	13 (4 – 68)
Bilateral cases, n (%)	11 (29%)
Prior PDT, n (%)	6 (12%)
Eyes with secondary CNV, n (%)	5 (10%)
Prior treatments, n (%)	Eplerenone (2, 5%)
Corticosteroid use, n (%)	9 (24%)
Hypertension, n (%)	4 (11%)
Diabetes mellitus, n (%)	2 (5%)
Sleep/psychiatric disorders, n (%)	1 (3%)

**Fig. 2:**
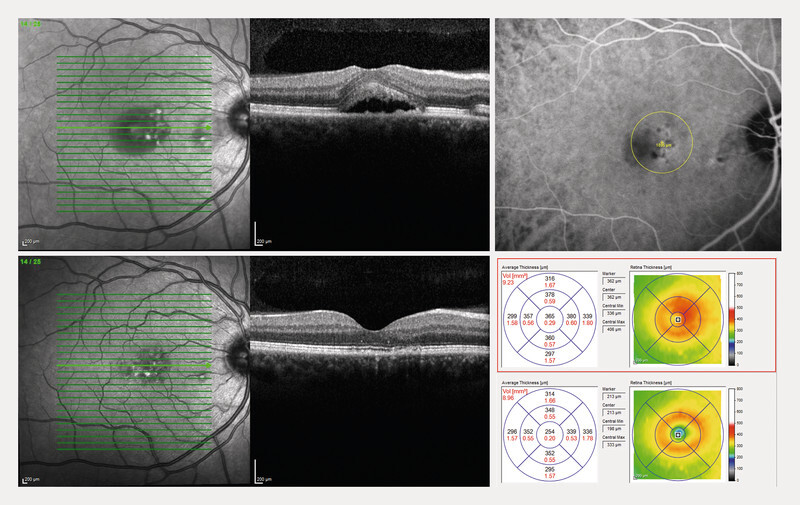
Multimodal imaging of a patient with central serous chorioretinopathy before and after photodynamic therapy (PDT). Top row (before PDT): Infrared fundus image with OCT scan lines (left), spectral-domain OCT showing subretinal fluid (center), and indocyanine green angiography (ICG) revealing choroidal hyperpermeability (right). Bottom row (6 months after PDT): Infrared image with scan lines (left), OCT demonstrating resolution of subretinal fluid (center), and retinal thickness maps (right) showing reduction in central macular thickness compared to baseline. The quantitative ETDRS grid analysis highlights decreased retinal thickness, consistent with anatomical improvement after treatment.

Analysis of the whole cohort revealed a significant improvement in best-corrected visual acuity with a p-value of 0.041 ( Fig. 1 a ). The mean visual acuity increased significantly after treatment, indicating functional improvement in a substantial portion of patients ( Table 2 ). Additionally, there was a highly significant reduction in central retinal thickness (CRT, p < 0.001) and subretinal fluid (SRF, p < 0.001), suggesting marked anatomical recovery ( Fig. 1 b ). The mean photodynamically treated spot diameter, as defined by the area of choroidal hyperpermeability on ICG, was 3.30 mm (SD: 1.24 mm), corresponding to an approximate treatment area of 8.6 mm². The treatment field size was adjusted individually based on the extent of choroidal hyperpermeability. The mean duration between initial diagnosis and PDT treatment was 13 months (with a total range of 1 to 68 months). The median follow-up after PDT was 2.1 months (interquartile range 2.0 – 2.4 months, range 0.4 – 14.0 months). Fig. 2 illustrates multimodal imaging of a representative patient showing the anatomical response to PDT. The PDT spot size was determined based on the full area of choroidal hyperpermeability on ICG angiography and was typically enlarged by a safety margin of approximately 500 µm to ensure complete coverage of the dysfunctional choroidal region. Post-treatment OCT and thickness maps demonstrate a marked reduction of subretinal fluid and central retinal thickness over a 6-month follow-up period.

**Table:** **Table 2** Outcomes of overall analysis with subgroup data.

Parameter	Mean Pre	SD Pre	Range Pre	Mean Post	SD Post	Range Post	p Value
Visual Acuity	0.58	0.28	0 – 1	0.65	0.33	0 – 1	0.041
CRT (µm)	352.6	107.5	204 – 737	281.2	92.2	141 – 502	< 0.001
SRF (µm)	157.0	93.2	18 – 393	76.8	85.4	0 – 300	< 0.001
Visual Acuity (Re-PDT)	0.52	0.29	0.1 – 1.2	0.65	0.37	0.1 – 1.2	0.250
CRT (Re-PDT) (µm)	336.5	85.6	204 – 507	291.5	100.9	179 – 468	0.031
SRF (Re-PDT) (µm)	164.90	99.5	18 – 393	113.29	101.7	0 – 300	0.031
Visual Acuity (CNV)	0.69	0.43	0.32 – 1.25	0.49	0.34	0.32 – 1.0	0,250
CRT (CNV) (µm)	372.80	154.2	260 – 644	373.4	80.9	286 – 502	0.625
SRF (CNV) (µm)	101.40	38.5	64 – 156	121.2	54.5	44 – 177	0.438

Regarding gender, the Mann-Whitney U test showed no significant differences between male and female patients concerning changes in visual acuity (p = 0.253), SRF (p = 0.882), or CRT (p = 0.990). The nearly identical mean ranks suggest that both sexes benefited similarly from PDT.

**Fig. 3:**
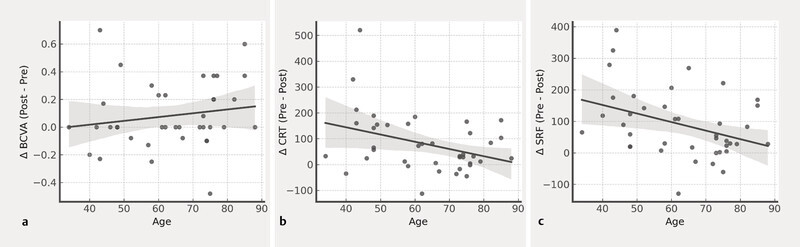
Correlation of patient age with visual acuity change, CRT reduction, and SRF reduction. Scatterplots showing a statistically significant inverse correlation between age and SRF reduction (r = − 0.315; p = 0.037), while trends in CRT (r = − 0.238; p = 0.120) and BCVA (r = 0.263; p = 0.101) did not reach significance. Linear regression lines with the corresponding regression equations and 95% confidence intervals are shown for each plot.

Correlation analysis between age and treatment effect showed a moderate negative association with SRF reduction (r = − 0.315; p = 0.037), indicating better anatomical outcomes in younger patients. Although the associations with visual acuity improvement (r = 0.263; p = 0.101) and CRT reduction (r = − 0.238; p = 0.120) were not statistically significant, there was a trend toward more favorable outcomes in younger individuals ( Fig. 3 ).

**Fig. 4:**
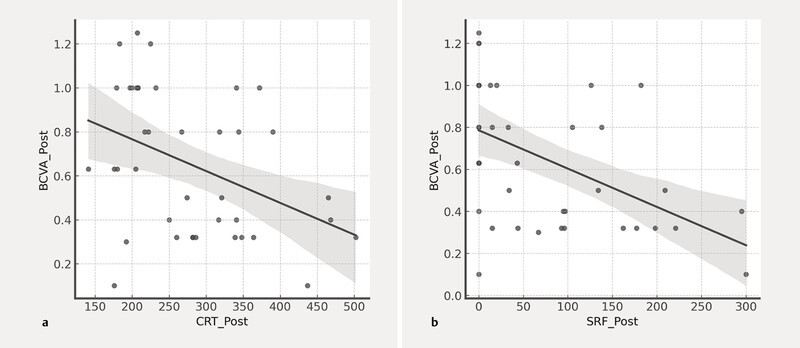
Correlation of post-treatment visual acuity with CRT and SRF. Scatterplots displaying significant negative correlations between visual acuity and both CRT (r = − 0.339; p = 0.033) and SRF (r = − 0.511; p < 0.001), indicating the prognostic value of OCT-derived morphological markers. Linear regression lines with the corresponding regression equations and 95% confidence intervals are shown for each plot.

Spearman correlation analysis also demonstrated a significant negative association between visual acuity and CRT (r = − 0.339, p = 0.033) as well as between visual acuity and SRF (r = − 0.511, p < 0.001), underscoring the functional relevance of morphological OCT parameters ( Fig. 4 ). A sensitivity analysis including only one eye per patient confirmed the robustness of the main results. Visual acuity improved significantly (p = 0.043), while both central retinal thickness and subretinal fluid showed highly significant reductions (CRT p < 0.001; SRF, p < 0.001). These findings demonstrate that the primary outcomes were not affected by potential intra-patient clustering.

## Exploratory Subgroup Analysis

Given the limited sample sizes, subgroup analyses were exploratory in nature and should be interpreted as trend-level findings rather than confirmatory results.

### Re-PDT (n = 6)

In patients who received repeated PDT, no statistically significant improvement in visual acuity was observed (p = 0.250, supplementary Figure 1a), although several cases showed functional gains. However, significant reductions in both CRT (p = 0.031) and SRF (p = 0.031) were documented (supplementary Figure 1b).

### CNV Subgroup (n = 5)

In patients with secondary choroidal neovascularization (CNV), PDT showed no significant therapeutic effect regarding visual acuity (p = 0.250, supplementary Figure 2a) or morphological parameters (CRT: p = 0.625; SRF: p = 0.438; supplementary Figure 2b). In three cases, visual acuity even declined. These findings should therefore be interpreted as exploratory trend-level results due to the small sample size.

These results suggest that patients with CNV may not benefit from PDT to the same extent as those with classic cCSC without a vascular component.

## Discussion

This retrospective analysis provides insights into the clinical efficacy of PDT in the treatment of cCSC at a single center. The findings demonstrate a significant reduction in CRT and subretinal fluid SRF, as well as a functional improvement in visual acuity across the overall cohort. These outcomes are consistent with previous studies and reinforce half-dose PDT as an effective therapeutic option for cCSC 12 , 13 , 14 , 15 , 16 .

Recent systematic reviews and international consensus guidelines have further strengthened the evidence base for half dose PDT in cCSC. A 2025 Cochrane network meta-analysis comprising 67 randomized controlled trials with 4015 participants found no clear superiority among treatment modalities but ranked half-dose PDT, eplerenone, and nutritional supplements as the highest-performing interventions for improving visual acuity–although the overall certainty of evidence remained low to moderate 17 . In parallel, the international consensus statement of the Asia-Pacific Vitreo-retina Society reaffirmed half dose PDT as the most evidence-based first-line therapy for cCSC, showing superior anatomical and functional outcomes compared with subthreshold micropulse laser therapy and mineralocorticoid receptor antagonists 2 . Novel anti-VEGF agents such as faricimab, are currently under investigation but have not yet demonstrated efficacy in typical cCSC. Collectively, these analyses and consensus data emphasize the continuing key role of half-dose PDT as the reference treatment while highlighting the need for high-quality comparative studies and individualized treatment strategies.

Subgroup analysis showed that patients receiving Re-PDT experienced notable morphological improvements, although no improvement in visual acuity was observed. This may suggest that prolonged disease duration leads to irreversible structural damage, thereby limiting functional recovery. Other studies have similarly identified baseline SRF levels and timing of intervention as key predictors of visual outcomes 18 . In our cohort, the functional response was less pronounced than the anatomical changes, with BCVA showing only a modest but statistically significant improvement after PDT.

In patients with secondary CNV, PDT did not yield a significant therapeutic effect. In fact, visual acuity deteriorated in several cases. These findings imply that patients with a vascular component may not benefit from PDT to the same extent as those with typical cCSC, even when adjunctive anti-VEGF therapy was administered. Overall, anti-VEGF agents such as ranibizumab or aflibercept may be more effective in these cases, as they have demonstrated superior fluid control and visual stabilization in randomized trials 19 , 20 . Careful differentiation using multimodal imaging is therefore essential.

Furthermore, the analysis revealed a significant negative correlation between visual acuity and the morphological OCT parameters CRT and SRF. Higher CRT and SRF values were associated with poorer visual acuity, underscoring the prognostic relevance of these structural biomarkers in cCSC.

The limitations of this study primarily include its retrospective design, potential selection bias, lack of standardized follow-up intervals, and the small number of cases in the subgroups. Accordingly, all subgroup analyses should be regarded as exploratory and indicative rather than confirmatory. Due to its retrospective nature, causal relationships cannot be established, and all associations should be interpreted with caution. Furthermore, the sample size of this retrospective study was constrained by supply shortages of verteporfin (Visudyne), which affected PDT availability throughout the study period. Recurrent supply shortages and production issues in Europe led to delays or limited use of PDT in many centers, including our center, Hanusch Hospital, Vienna. While these external factors do not affect treatment efficacy, they do impact the practical implementation and planning of studies under real-world conditions.

Because both eyes were included in some patients, intra-subject correlation could have influenced variance estimates. However, a sensitivity analysis based on one eye per patient confirmed consistent and statistically significant results (BCVA p = 0.043; CRT and SRF p < 0.001), supporting the robustness of the main findings.

## Clinical Implications

Photodynamic therapy remains the first-line treatment for chronic CSC with persistent subretinal fluid beyond three months or insufficient response to observation. Early treatment is advisable in patients with preserved visual acuity and without advanced photoreceptor or RPE damage. Repeat PDT can be considered for recurrent or refractory cases with persistent choroidal hyperpermeability. Conversely, patients with secondary choroidal neovascularization should be carefully evaluated for combination or alternative therapy using anti-VEGF agents. In the context of current verteporfin shortages, prioritizing treatment for patients with the greatest risk of irreversible visual decline and considering subthreshold micropulse laser as a temporary alternative may help to optimize patient care in real-world settings. Overall, individualized treatment planning based on disease chronicity, lesion morphology, and visual potential is essential to optimize outcomes.

The clinical implementation of PDT is currently constrained by recurrent verteporfin (Visudyne) shortages in Europe and complex reimbursement processes for off-label use in cCSC 21 . During supply shortages, treatment should be prioritized for patients at high risk of irreversible vision loss, while subthreshold micropulse laser may serve as a temporary alternative. Stable verteporfin availability and simplified reimbursement pathways remain essential to ensure guideline-based care.

## Conclusion

In conclusion, the results confirm the clinical relevance of PDT in the management of cCSC. Future prospective, multicenter studies with defined control groups and standardized treatment intervals are desirable to further strengthen the evidence base 22 .

Conclusion Box **Already known:**

- Chronic CSC is associated with persistent subretinal fluid, fluctuating visual function, and progressive photoreceptor/RPE damage.
- Photodynamic therapy (PDT) is an established treatment and has shown superior anatomical efficacy compared with laser-based alternatives in randomized trials.
- It remained unclear to what extent half-dose PDT provides functional benefits and how reproducible these effects are in real-world clinical practice.

**Newly described:**

- This study demonstrates significant anatomical and functional improvement after half-dose PDT in a real-world cohort of chronic CSC patients.
- The exploratory subgroup analysis highlights limited functional benefit in cases with prolonged disease duration or secondary CNV.
- The findings emphasize the importance of early intervention and provide further evidence supporting half-dose PDT as a first-line therapy in chronic CSC.

## Note

The authors used AI-based language assistance for grammar refinement.
