# Supplementary material for: Efficacy of Photodynamic Therapy in Chronic Central Serous Chorioretinopathy: A Retrospective Analysis at Hanusch Hospital, Vienna
Source: Klin Monbl Augenheilkd. 2026 Feb 10;243(7):759–66. doi: 10.1055/a-2772-8827 (PMC13384739; doi:10.1055/a-2772-8827)
Supplement: Supplementary file 1 — Supporting Information [file 10-1055-a-2772-8827-sup_kl3307.pdf]

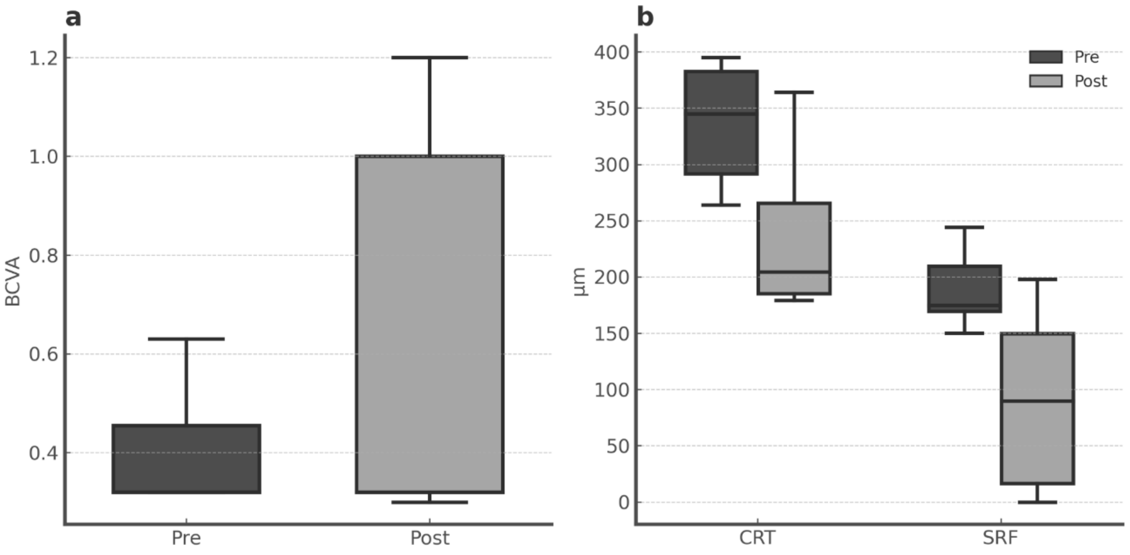

Supplementary Figure 1:

(a) Visual acuity before and after repeat PDT (Re-PDT).

Boxplot displaying changes in BCVA in eyes undergoing Re-PDT. Although some patients showed improvement, the change was not statistically significant (p = 0.109). (b) CRT and SRF before and after Re-PDT.

Boxplots demonstrating significant reductions in both CRT and SRF after Re-PDT (p = 0.028 for both parameters), suggesting anatomical benefit even with repeated treatment.

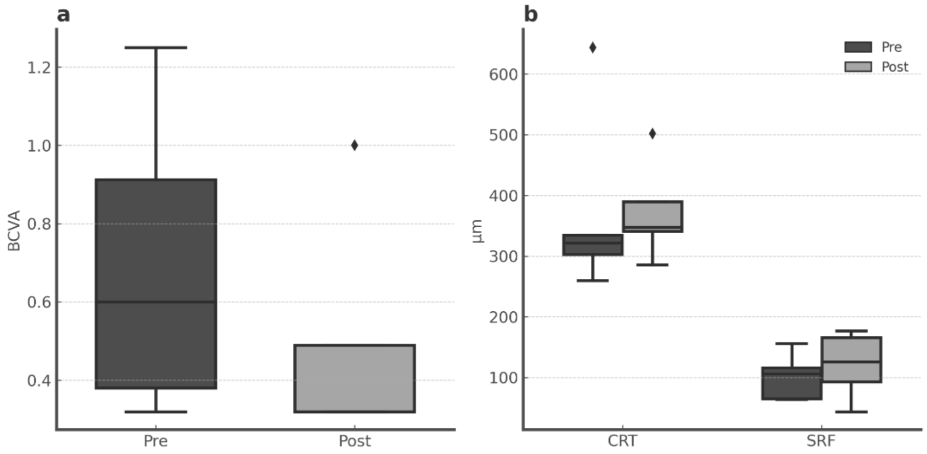

Supplementary Figure 2:

(a) Visual acuity before and after PDT in patients with secondary choroidal neovascularization (CNV).

Boxplot showing a non-significant decrease in BCVA post-treatment (p = 0.109), highlighting the limited functional effect of PDT in this subgroup.

(b) CRT and SRF before and after PDT in the CNV subgroup.

Boxplots indicating no significant change in CRT (p = 0.500) or SRF (p = 0.345), supporting the limited morphological effect of PDT in CNV-associated cCSC.
